# A Generalizability Analysis of the Mobile Phone Addiction Tendency Scale for Chinese College Students

**DOI:** 10.3389/fpsyt.2019.00241

**Published:** 2019-04-12

**Authors:** Guangming Li, Jinyan Xie, Like An, Guiyun Hou, Hu Jian, Weijun Wang

**Affiliations:** ^1^Guangdong Key Laboratory of Mental Health and Cognitive Science, Center for Studies of Psychological Application, School of Psychology, South China Normal University, Guangzhou, China; ^2^School of Public Finance and Public Administration, Jiangxi University of Finance and Economics, Nanchang, China; ^3^Department of Psychology, Clinical and Research Institute on Addictions, University at Buffalo, State University of New York, Buffalo, NY, United States

**Keywords:** mobile phone addiction, generalizability theory, generalization coefficient, dependability index, contribution ratio

## Abstract

College students’ mobile phone addiction is negatively associated with physical and mental health and academic performance. Many self-made questionnaires are currently being administered to Chinese college students to evaluate the mobile phone addiction tendency. Using the univariate generalizability theory and multivariate generalizability theory, this study investigated the psychometric properties and the internal structure of the Mobile Phone Addiction Tendency Scale (MPATS), the most widely used survey questionnaire assessing the status of Chinese college students’ mobile addiction. Data were a sample of 1,253 college students from the southwest of China. Primary analytic approaches included the generalizability design of univariate random measurement mode *p* × (*i*:*h*) and multivariate random measurement mode *p*˙ × *i°*. Results showed that the variance component of the participants and the variation related to the participants explained most of the variation of the scale, while the variance component of the items was small, and the generalizability coefficient and dependability index of the scale were 0.88 and 0.85. In the multivariate generalizability analysis, the variance component of the participants and the variation related to the participants accounted for most of the variation of the scale and the variance component of the items was small. The generalizability coefficients of withdrawal symptoms, salience, social comfort, and mood changes were 0.64–0.80, and the dependability indexes were 0.63–0.77. However, the generalizability coefficient and reliability index of universe score were 0.91 and 0.90. In addition, the contribution ratio of the four dimensions to the universe score variance was different from the assignment intention of the initial scale. Recommendations were discussed on the improvement of the test reliability for each dimension.

## Introduction

Mobile phone use among college students has become daily experiences—for the purpose of communication, entertainment, camera, calculation, reading, and document editing. Mobile phone use may make students’ everyday study, work, and daily lives more convenient, however, it may also bring about present and potential threats. College students who have been immersed in mobile phone use often show no interest in (returning to) the real world. It is extremely harmful to use mobile phone continuously for a long time—one may suffer from headaches, dizziness, body aches, numbness in hands and feet, dry eyes, and blurry eyes (1). The abuse of mobile phone may also cause sleep problems (2), symptoms such as loneliness, anxiety, and depression (3–5), and negatively impact academic performance (6). Like internet-related disorders, excessive usage and the abuse of mobile phone may cause a risk of behavior addiction (7), because excessive use of mobile phone (and internet) is often associated with a number of primary addiction symptoms, such as apparent tolerance, withdrawal, loss of control, craving, cognitive salience, or emotional regulation [e.g., Refs. (8, 9)]. Mobile phone use-related behavioral addiction may involve both dysfunctions (e.g., serious adverse effects on daily life) and continuation and stability of these dysfunctional behaviors.

Mobile phone addiction has been a scientific research focus. Han and Qi (10) argued that mobile phone addiction should be defined as including the abuse of mobile phones, having a serious effect on a person’s life, work, or study, and having the feeling of a series of discomforts when the phone stops working or is not in use. Griffith (11) suggested that mobile phone addiction is a nonbiochemical behavior addiction, a human–computer interaction. Heavy mobile phone users may use mobile phones as a social strategy to avoid facing negative life events (e.g., unemployment, coping with trauma) or social anxiety (12, 13). They may also desire for some kind of achievement, for example, in excessive participation in dysfunctional games (e.g., to be a powerful avatar or a guild master) (8). Indeed, increased time spent on a particular behavior on mobile phone can be driven by multiple motivations. Young people may use mobile phone to develop new abilities and skills and enhance self-efficacy and self-esteem (8). However, we believe that mobile phone addiction involves one’s dependence of excessive use of mobile phones, a strong physiological experience, and behavioral discomfort. Mobile phone addiction has not yet been treated as a clinical psychological disorder.

The literature has documented several questionnaires assessing (dimensions of) mobile phone addiction in Chinese adolescents and college students. For example, Xu et al. (14) developed the College Student’s Mobile Phone Dependency Scale, which included a total of 13 projects to capture four dimensions: behavioral (psychological) tolerance, behavioral (psychological) withdrawal, social function, and physiological response. The reliability of that scale was low, and the sample capacity was too small. Su and colleagues (15) developed the College Student Mobile Phone Dependence Scale, with six dimensions (withdrawal behavior, prominent behavior, social comfort, negative influence, App usage, and App update) containing 22 projects in total, but the projects distributed in each dimension were not sufficiently balanced. 

Most published studies that examined Chinese young people and their mobile phone addiction have used the Mobile Phone Addiction Tendency Scale (MPATS) (16). The MPATS emphasized the internal processing activities and subjective experiences among social interactions of mobile phone users including four dimensions: withdrawal symptoms, salience, social comfort, and mood changes. These studies reported a 0.83 Cronbach coefficient of the total scale and the alpha coefficients of the four subscales ranged from 0.55 to 0.80. In addition, the test–retest reliability of the total scale was 0.91, and the test–retest reliability of the four subscales was between 0.75 and 0.85, and the fitting indicators were good.

The measurement evaluation index of the MPATS, however, has been based on the classical test theory (CTT). Analyses of parameters (such as reliability, validity, difficulty, and discrimination of the project) have been heavily dependent on selected college students in each research institute. Therefore, results from these analyses could only evaluate that survey and the information contained in the questionnaire should not be promoted. In addition, CTT merely considers the general error, but does not identify various sources and the magnitude of errors that may affect the measurement object, so the reliability cannot be accurately estimated and the scheme for reducing the error cannot be proposed. The concept of reliability in CTT only applies to a standardized normative reference test, which has limitations in the estimation methods (17). Also, the assumption of its strict parallel test is difficult to meet in practice. In order to solve these problems, Cronbach et al. (18) proposed the generalizability theory (GT), which introduces experimental designs and analysis of variance to decompose various types of error generated in the measurement process based on CTT, and thereby allows to calculate the reliability and validity of the test more accurately.

GT uses the idea of experimental design to analyze the important variance sources that influence participants’ scores and applies the ANOVA analysis techniques to estimate the effect of various sources of variation on the total variation of scores. According to different research purposes, the proportion of the measurement facets in the total variation of the test can then be examined (19). The generalizability coefficient and dependent index in GT can distinguish the relative error and the absolute error in the measurement process and represent the reliability concept of measurement. GT is divided into univariate GT (UGT) and multivariate GT (MGT). In the UGT, the variation of the measurement object is represented by the universe score of a variable, whereas the variation of the measurement object is composed of the universe scores of multiple variables in the MGT. Using the UGT to analyze a scale’s measurement properties, the concept is easy to understand and the operation is simple. It can estimate the variance component (VC) of the measurement object (scale score) and measurement facets and its proportion, the generalizability coefficient and dependent index of the scale score, but it cannot get the corresponding indicators in each dimension. MGT is more advantageous when dealing with measurement problems that have multiple potential capability factors and there are correlations among these factors (20). When MGT evaluates the measurement properties of the scale, it can not only estimate the VCs, generalization coefficients, and dependent indexes of the scale’s total scores, but also estimate the VCs, covariance components, and generalizability of each dimension. The internal structure of the scale can be analyzed in depth through the covariance components of different dimensions, that is, the rationality of the dimension of the scale. Therefore, for the evaluation of the scale, UGT can explore its general psychometric attributes and MGT can analyze the relationship between various dimensions so as to further investigate the internal structure of the scale and the consistency of the scores in different dimensions.

The measurement context relationship in GT consists of the measurement object and the measurement facet. There are three main measurement modes: random measurement mode, fixed measurement mode, and mixed measurement mode. If the conditional sample on the measurement facet is randomly sampled from the universe of the admissible observations, the measurement mode is a random measurement mode, and the facet is a random facet. If the conditional sample on the measurement facet is fixed, it is a fixed measurement mode (that is, the standardized test in CTT) and the facet is a fixed facet. However, if there are both random facets and fixed facets in the measurement process, it is a mixed measurement mode (19). According to the measurement tools of mobile phone addiction prepared by previous researchers, it can be seen that the measurement dimensions of different scales were not the same and could be regarded as being randomly sampled from the universe of admissible observations of mobile phone addiction dimensions. Therefore, this paper selected the random measurement mode.

In summary, the present study used UGT and MGT to evaluate the psychometric attributes of the MPATS and examined the internal structure of the four dimensions of the scale. It was expected that the present study would improve the measurement accuracy and structure of the scale and provide references for the application of this scale in practice. We provided the following four hypotheses:

H1: The VC of the participants would be large, while the VCs of the dimensional effect, the effect of items nested within the dimension, as well as the interaction term between the participant and the dimension would be small; in addition, the VC of the mixed effect of pi:h would be large;
H2: The VC of the absolute error and the relative error of the scale’s universe score would be small, whereas the generalizability coefficient and the dependent index of the scale’s universe score would be large;
H3: The VCs of the items in each dimension would be small; in terms of the four dimensions, the covariance components of withdrawal symptoms, salience, and mood changes on participants’ effect would be large, whereas the covariance component of social comfort would be small; in addition, the VCs of the four dimensions in the interaction effects between the participants and the items would be large; and
H4: The generalizability coefficients and dependent indexes of withdrawal symptoms, salience, and social comfort would be large, while the generalizability coefficient and dependent index of mood changes would be small.

## Methods

### Participant

Data included 1,253 college students (59.0% females; 60.8% Han nationalities, and 39.2% ethnic minorities) from three universities in the southwest of China. Students completed the MPATS questionnaire in class using a paper–pencil version in May 2017. This survey study was approved by the university research ethics board (institutional review board).

### Assessments

Mobile phone addiction was measured using the MPATS (16). The items examined perceptions of college students’ withdrawal symptoms (six items; e.g., “Mobile phones are a part of my life. I feel like I’ve lost something once I will have to limit the time I spend on my mobile phone”), salience (four items; e.g., “I often have the illusion that ‘my cell phone rings or vibrates’”), social comfort (three items; e.g., “I feel more confident to communicate with others using my mobile phone (than to talk face to face)”), and mood changes (three items; e.g., “When the phone is not connected to the line or receives no signals, I will become anxious and get angry”). All 16 items were rated on 5-point scales ranging from 0 (very inconsistent) to 4 (very consistent). Items were summed to create a composite score for each participant, with higher scores indicating greater perceived tendency of mobile phone addiction. The MPATS had a good internal consistency (♐*α* = 0.90; the alpha coefficients of withdrawal symptoms, salience, social comfort, and mood changes were 0.80, 0.75, 0.72, and 0.64, respectively).

The four domains or role categories were not intended to represent orthogonal subscales; rather they were identified for the purpose of enhancing the content validity of the measurement (16). The present study aimed to investigate the precision of the MPTAS using a generalizability analysis of the domain measures. This approach identifies and quantifies sources of variance contributing to errors in measurement.

### Measurement Design

The two-facet unbalanced design *p* × (*i*:*h*) in the UGT and the one-facet multivariate design *p*˙ × *i*° in the MGT were used respectively to analyze the MPATS data in college students. Participant *p* is the measurement object and items *i* of each dimension are the measurement facets and the measurement dimension *h* is also the measurement facet in this study. In the two-facet unbalanced design, the facet *h* is a random measurement facet that contained four levels (i.e., withdrawal symptoms, salience, social comfort, and mood changes). In the one-facet multivariate design, the facet *h* is a fixed multivariate variable that is divided into four subvariables (i.e., four measurement sub-objects: withdrawal symptoms, salience, social comfort, mood changes). In addition, a solid circle superscript in the MGT indicates that the measurement object or facet crosses a fixed multivariate variable, whereas a hollow circle superscript indicates that the measurement object or facet is nested in a fixed multivariate variable, thus the facet *i* is nested in the dimension *h*. The two generalizability designs used urGENOVA and mGENOVA software packages for data processing and statistical analyses.

## Results

### Descriptive

The minimums, maximums, means, standard deviations, and bivariate correlations among the four dimensions of the MPTAS are presented in **Table 1**.

**Table 1:** Minimums, maximums, means, standard deviations, and bivariate correlations among the four dimensions.

	Descriptive statistics					Correlations			
Variable	N	Min	Max	Mean	SD	1	2	3	4
1. WS	1,253	1.00	5.00	2.81	0.78	–			
2. S	1,253	1.00	5.00	2.49	0.82	0.748**	–		
3. SC	1,253	1.00	5.00	2.56	0.82	0.549**	0.577**	–	
4. MC	1,253	1.00	5.00	2.49	0.84	0.648**	0.688**	0.533**	–

WS, withdrawal symptoms; S, salience; SC, social comfort; MC, mood changes.

**Correlation is significant at the 0.01 level.

### The Univariate Generalizability Results of the Mobile Phone Addiction Tendency Scale

The measurement mode is the two-facet unbalanced design, because the number of items for the four dimensions of the MPATS is different (i.e., there are six items for withdrawal symptoms, four items for salience, three items for social comfort, and three items for mood changes). The item *i* is nested in the dimension *h* and crosses the participant *p*. There are five VCs, namely, *p*, *h*, *i:h*, *ph*, and *pi:h*.

#### The Results of Univariate Generalizability Theory G-Study

Results of the two-facet unbalanced design G-study are displayed in **Table 2**. The VC of the participants was 0.42082, accounting for 33.471% of the total variation of the scale, which indicates that there were individual differences in scoring the scale.

**Table 2:** Estimated G-study variance components for the MPATS, based on *p* × *(i:h)* design (N = 1,253).

Source	*df*	*T*	*SS*	*MS*	*VC*	*PTVC*
Participant (*p*)	1,252	147,734.1	9,622.373	7.6856	0.42082	0.33471
Dimension (*h*)	3	138,544.7	432.9229	144.3076	0.00972	0.00773
Item within dimension (*i:h*)	12	139,707.1	1,162.408	96.8673	0.07676	0.06105
Participant by dimension (*ph*)	3,756	151,632	3,464.952	0.92251	0.06002	0.04774
Residuals (*pi:h*)	15,024	163,160	10,365.59	0.68994	0.68994	0.54876

VC is the estimated variance component. MPATS, Mobile Phone Addiction Tendency Scale.

PTVC (proportion total variance component) is the ratio of each effect to the total variation.

The VCs of the dimensional effect, the effect of items nested within the dimension, as well as the interaction between the participant and the dimension were all less than 0.08, indicating no significant effects on the total variation of the scale. The VC of the mixed effect of *pi:h* accounted for 54.876% of the total variation of the scale, suggesting that the mixed effect of the participants crossing items and nested in the dimensions had a greater impact on the total variation of the scale. However, the two-facet unbalanced design in UGT did not determine which dimension’s variance had a greater impact on the scale. Therefore, we used the MGT to further explore these VCs.

#### The Results of Univariate Generalizability Theory D-Study 

We performed the two-facet unbalanced design of generalizability analyses using the urGENOVA software, which could not perform subsequent analyses with D-study. According to the formulas recommended by Brennan (20; see below), the VCs of the absolute error and the relative error scores can be calculated, and thus, the generalizability coefficient and dependent index can be obtained. The results of D-study showed that the VC of the absolute error and the relative error were 0.07157 and 0.05953, respectively. In addition, the generalizability coefficient and the dependent index were 0.87606 and 0.85466, respectively, suggesting that the MPTAS could be used as a standard reference test and also a norm reference test. However, the generalizability coefficient and dependent index of each dimension could not be calculated, and it was still necessary to use MGT to continue the analyses.

$$\text{Absolute error: }\sigma^{2}(\Delta )=\frac{\sigma^{2}(h)}{{\overset{⌣}{n}}_{h}}+\frac{\sigma^{2}(i\text{ :}h)}{n_{i+}}+\frac{\sigma^{2}(ph)}{{\overset{⌣}{n}}_{h}}+\frac{\sigma^{2}(pi\text{ :}h)}{n_{i+}}$$

$$\text{Relative error: }\sigma^{2}(\delta )=\frac{\sigma^{2}(ph)}{{\overset{⌣}{n}}_{h}}+\frac{\sigma^{2}(pi\text{ :}h)}{n_{i+}}$$

$$\text{Generalizability coefficient: E}\rho^{2}=\frac{\sigma^{2}(p)}{\sigma^{2}(p)+\sigma^{z}(\delta )}$$

$$\text{Dependent index: }\Phi =\frac{\sigma^{2}(p)}{\sigma^{2}(p)+\sigma^{z}(\Delta )}$$

### The Multivariate Generalizability r Results of the MPATS

In the one-facet multivariate design *p*˙ × *i*˚, the participant *p* crosses the dimension *h*, and the item *i* is nested in the dimension *h*. The VC design is *p* × *i* and the covariance component is *p*.

#### The Results of Multivariate Generalizability Theory G-Study 

According to the research design of the four-factor dimension model, the estimation matrix of the variance and covariance component of the interaction *(p)*, topic *(i)*, and the interaction between the subject and the item *(pi)* can be obtained on all four factor dimensions (withdrawal symptoms, prominent behavior, social comfort, and mood change) in the G-study stage. Results are shown in **Table 3**. The VCs of the items in each dimension were less than 0.14, showing that the items had no significant impact on the total variation of the scale. In terms of the four dimensions, the covariance components of withdrawal symptoms, salience, and mood changes on participants’ effects were relatively large (0.47569, 0.42363, and 0.47098) and the covariance components of social comfort and the other three dimensions were relatively small (0.35362, 0.38751, and 0.36865). The correlation coefficients among all dimensions were all above 0.78, which indicates that it may be feasible to synthesize the scores into a universe score of mobile addiction. However, whether the four dimensions could be synthesized to the total score of the scale would depend on the results of D-study. Indeed, the VC of the participant and the effects related to the participant accounted for most of the total variation of the scale. 

**Table 3:** Estimated variance and covariance components for *p*˙ × *i*° design in MGT G-study for the four dimensions of the MPATS (N = 1,253).

	Withdrawal symptoms	Salience	Social comfort	Mood changes
Participant ( *p*)	**0.48574**	0.96471	0.72322	0.90529
	0.47569	**0.50056**	0.78292	0.99147
	0.35262	0.38751	**0.48940**	0.78486
	0.42362	0.47098	0.36865	**0.45080**
Items (*i *)	**0.13089**			
		**0.06539**		
			**0.00380**	
				**0.03143**
Participant by items ( *pi *)	**0.72897**			
		**0.66060**		
			**0.56710**	
				**0.75920**

MGT, multivariate generalizability theory. Diagonal elements are estimated variance components and are presented in bold type. The lower diagonal elements are covariances, and the upper diagonal elements are correlations.

The VCs of the four dimensions in the interaction effects between the participants and the items were relatively large, indicating that the interaction effects between the participants and the items had a greater impact on the total variation of the scale. Whether the test could be used as a norm reference test or a standard reference test would require further reference to the results of D-study.

#### The Results of Multivariate Generalizability Theory D-Study

According to the variance and covariance matrix estimated by G-study, the global score of the four factors and the VC of the corresponding error can be estimated in the D-study stage, and then the estimated value of the generalized coefficient, the reliability index, the relative signal-to-noise ratio, and the absolute signal-to-noise ratio were obtained. Results were displayed in **Table 4**. The generalizability coefficient (0.91059) and the dependent index (0.90213) of the scale’s universe score were large, significantly larger than each dimension’s coefficient. Whereas the relative error variance (0.04295) and the absolute error variance (0.04745) of the scale’s universe score were significantly lower than the error variance of each dimension. The generalizability coefficients and dependent indexes of withdrawal symptoms, salience, and social comfort were all greater than 0.7 but less than 0.8. However, the generalizability coefficient and dependent index of mood changes were small, only greater than 0.6. The generalization coefficient and reliability index of mood change can be improved by increasing the question items or improving the quality of the question items. As for the total scale, it is feasible to synthesize all dimensions’ scores to improve the reliability of the scale.

**Table 4:** Estimated MGT D-study statistics for the MPATS.

Index	Withdrawal symptoms	Salience	Social comfort	Mood changes	Universe score
USV	0.69695	0.70750	0.69957	0.67141	0.43740
REV	0.34856	0.40639	0.43478	0.50306	0.04295
AEV	0.37856	0.42603	0.43623	0.51337	0.04745
EVM	0.14933	0.12992	0.04251	0.10507	0.00489
GC	0.79992	0.75192	0.72137	0.64046	0.91059
DI	0.77218	0.73390	0.72003	0.63107	0.90213
S/NR	3.99804	3.03095	2.58898	1.78132	10.18405
S/NA	3.38945	2.75793	2.57176	1.71051	9.21776

USV is the universe score variance; REV is the relative error variance; AEV is the absolute error variance; EVM is the error variance for mean; GC is generalizability coefficient; DI is dependent index; S/NR is signal/noise-relative; S/NA is signal/noise-absolute.

### The Contribution Ratio of Each Dimension to the Scale Universe Score

The weight coefficient (w) is the percentage of number of items in each dimension as a percentage of the total test items. As shown in **Table 5**, the weight coefficient of withdrawal symptoms, salience, social comfort, and mood change were 0.375, 0.25, 0.1875, and 0.1875, respectively. Withdrawal symptoms and salience contributed more to the universe score variance than their scores on the scale, whereas social comfort and mood changes were lower than their score ratios on the scale. Withdrawal symptoms contributed the most to the universe score variance, followed by salience, mood changes, and social comfort. Withdrawal symptoms contributed the most to the relative error and the absolute error, followed by salience, mood changes, and social comfort.

**Table 5:** The contribution ratio of each dimension to the scale universe score.

Index	WS	S	SC	MC
Number of dimension	6	4	3	3
Total score of each dimension	30	20	15	15
w	0.375	0.25	0.1875	0.1875
Score ratio the for each dimension (%)	0.375	0.25	0.1875	0.1875
Contributions to the universe score variance (%)	38.29	26.55	16.72	18.44
Contributions to the relative error variance (%)	39.78	24.03	15.47	20.71
Contributions to the absolute error variance (%)	42.47	23.91	14.10	19.53

WS, withdrawal symptoms; S, salience; SC, social comfort; MC, mood change.

### The Results of D-Study to Improve Measurement Reliability

**Table 6** presents the results of D-study on the changes in the number and reliability of the four dimensions of the scale. As the number of items in each dimension increased, the reliability of the dimensions also increased. When the items of withdrawal symptoms, salience, social comfort, and mood changes increased to 7, 6, 5, and 7, respectively, the reliability of each dimension was significantly improved, and the measurement indicators were optimal.

**Table 6:** The results of D-study for the number of the dimensions and the change of reliability.

Dimension	Index	1	2	3	4	5	6	7	8
WD	*Ep* *^2^*	0.39988	0.57131	0.66656	0.72718	0.76914	0.79992	0.82346	0.84204
	Φ	0.36098	0.53048	0.62890	0.69322	0.73853	0.77218	0.79816	0.81882
S	*Ep* *^2^*	0.43109	0.60246	0.69449	0.75192	0.79117	0.81970	0.84137	0.85840
	Φ	0.40810	0.57965	0.67410	0.73390	0.77515	0.80533	0.82837	0.84653
SC	*Ep* *^2^*	0.46323	0.63316	0.72137	0.77538	0.81185	0.83813	0.85797	0.87348
	Φ	0.46157	0.63161	0.72003	0.77422	0.81083	0.83723	0.85716	0.87274
MC	*Ep* *^2^*	0.37256	0.54287	0.64046	0.70371	0.74804	0.78083	0.80607	0.82609
	Φ	0.36313	0.53278	0.63107	0.69518	0.74032	0.77381	0.79965	0.82019

The underlined value indicates the original index of the dimension, Ep^2^ is the generalization coefficient, and Φ is the reliability index. WD, withdrawal symptoms; S, salience; SC, social comfort; MC, mood changes.

## Discussions

This study used GT of modern measurement theory to evaluate the measurement attributes of the MPATS using UGT and MGT designs, which is similar to the study of structural validity in CTT. We aimed to investigate the internal structure of the scale in more depth and identify its defects and deficiencies.

Results of UGT and MGT G-study showed that the VCs of the participants and the VCs related to the participants accounted for most of the total variation of the scale. There were individual differences among participants in scoring the mobile phone addiction scale. Based on the UGT analyses, the variance of the dimension’s effect, the effect of the items nested in the dimension, and the interaction effect between the participant and the dimension were all small. These effects had little influence on the total variance of the scale. H1 was supported. The MGT analyses also showed that the VCs of the four dimensions’ items were small, which further indicated that the items were not the main source of measurement error.

Results of UGT D-study showed that the absolute error and the relative error of the scale were small, and generalizability coefficient and dependent index were both large (H2). Additionally, the MGT D-study showed that the generalizability coefficient and dependent index of the scale’s universe score were higher than the four dimensions’ generalizability coefficient and dependent index and the error variance of the scale’s universe score was lower than the error variance of each dimension. These results indicated that it is feasible to synthesize the scale’s universe, which would effectively improve the reliability of the scale. The generalizability coefficient and dependent coefficient of the scale were high, suggesting that the scale can be used as a norm reference test or a standard reference test. However, each factor should not be used alone as a test. This is also consistent with the findings of several other studies (21–23). The whole measurement scale was tested with a good reliability. Whether the MPATS should be used as a norm-referenced test for relative decision-making or a standard reference test for absolute decision-making will necessarily rely on the purpose of studies. If a study intends to test and distinguish the status of mobile phone addiction among college students, the MPATS should be treated as a norm-referenced test to interpret the results of the measurement. However, if a study aims to understand the actual situation of individual mobile phone addiction, the scale should be used as a standard reference test to interpret the results in absolute terms. In addition, the MGT D-study also showed that the generalizability coefficient and dependent index of withdrawal symptoms, salience, and social comfort were better, compared with the dimension of mood changes, but all were less than 0.8. The VC of the relative error and the absolute error of mood changes were largest among the four dimensions. The reliability of the four dimensions should be improved, especially that of the mood changes. Results were consistent with H4.

In the process of preparation for the test, increasing the number of test items can effectively improve the reliability. However, CTT cannot meet the need to add items to increase the reliability standards for the test. Generalizability theory (GT) can consider the sources of multiple measurement errors to find the optimal measurement conditions (20), and D-study can also provide a reference for deciding on the number of test items so as to meet the psychometric scale’s reliability requirements. Results of D-study also showed that with the increasing of the number of items in each dimension, the reliability of dimensions also increased. The generalizability coefficient and dependent index in GT were typically greater than 0.8 to indicate good test reliability (24). The original indicators (generalizability coefficient and dependent index) of each dimension did not reach 0.8, and increasing the number of items can effectively improve the reliability of each dimension. When the number of items of the four dimensions was increased to 7, 6, 5, and 7, these indexes would be optimal.

In the G-study of UGT, the proportion of the VC of the *pi:h* mixed effect in the scale was as high as 54.876%, which indicated that there were individual differences in the mobile phone addiction situation when participants rated the items of the four different dimensions. This mixed effect had the largest impact on the total variation of the scale (H1). Results of MGT D-study showed that the contributions of each dimension to the universe score of the scale were ranked as follows: withdrawal symptoms, salience, mood changes, and social comfort. The findings are also consistent with previous studies (14, 15, 25, 26). Many self-made measurement scales assessing college students’ mobile phone addiction included similar subscales such as withdrawal symptoms and salience. Mobile phones are the center of the daily lives for those students who are addicted to mobile phones. They may become anxious, and they may conduct some negative behaviors if they have no or limited access to mobile phones. On Chinese college campus, withdrawal symptoms and salience are key manifestations of students’ mobile addiction.

In addition, the contributions of each dimension to the universe score of the scale were different, and the contribution ratio of withdrawal symptoms and salience to the universe variance were higher than the proportion of scores in the scale, and social comfort and mood changes were lower. There were differences among the contribution ratio of the four dimensions to the universe variance and the intention of the scale when compiled. The college students’ mobile addiction tendency scale is not perfect in determining the proportion of each factor. It is possible that, in addition to increasing the number of items, to change the contribution of different dimensions to the global total variance would also improve the quality of test items for mood changes and social comfort.

With regard to the internal structure of the scale, results of MGT G-study showed that withdrawal symptoms, salience, and mood changes were related to the effect of the participants. Their covariance components were also large. Participants’ scores on withdrawal symptoms, highlighting behavior, and mood changes were more consistent with different mobile phone addiction propensities. Yet the covariance components of social comfort and the other three dimensions were small, which indicates that the degree of consistency of the participant’s mobile phone addiction propensity explained by social comfort and any other dimension was relatively low. The correlation coefficient among various dimensions reached a moderate correlation, indicating that these four dimensions were both related and independent. Therefore, the items of social comfort need to be improved. What’s more, the VCs of the four dimensions in the interaction effect between the participants and items were relatively large, showing that different participants responded to the same scale but the scores were not the same. This may be related to the relatively high proportion of minority college students in this test population. These results supported H3. Students of different nationalities (39.2% ethnic minorities in this study) may understand the scale differently, and they may also judge the personal mobile phone addiction tendency differently. Future study should examine ethnic differences in mobile phone addiction among college students.

## Conclusions

The two generalized designs (the two-facet unbalanced design and the one-facet multivariate design) indicated that the effects of the participate and the effects associated with the participate accounted for the majority of the total variation of the MPATS for Chinese college students, and the test items were not the main source of measurement errors. According to the generalizability coefficient and dependability index, the MPATS can be used either as a Norm Referenced Test or as a Criterion Referenced Test, but it depends on the research intention of the study that uses this scale. For the specific dimensions of the MPTAS, the reliability of the test can be improved by increasing the number of items in each dimension, and the contributions of mood changes and social comfort to the scale can also be improved by improving the quality of items.

## Author Contributions

All authors significantly contributed to the work and gave the final approval to the current version of the manuscript.

## Funding

This research was funded by grant no. 31470050 from the National Natural Science Foundation of China.

## Conflict of Interest Statement

The authors declare that the research was conducted in the absence of any commercial or financial relationships that could be construed as a potential conflict of interest.
